# Discharging Preterm Infants Home on Caffeine, a Single Center Experience

**DOI:** 10.3390/children7090114

**Published:** 2020-08-28

**Authors:** Cheng Ma, Denisse Broadbent, Garrett Levin, Sanjeet Panda, Devaraj Sambalingam, Norma Garcia, Edson Ruiz, Ajay Pratap Singh

**Affiliations:** 1 Paul L. Foster School of Medicine, Texas Tech University Health Sciences Center El Paso 4800, Alberta Avenue, El Paso, TX 79905, USA; cheng.ma@ttuhsc.edu (C.M.); Denisse.Broadbent@ttuhsc.edu (D.B.); Garrett.Levin@ttuhsc.edu (G.L.); Sanjeet.Panda@ttuhsc.edu (S.P.); jayvirujay@gmail.com (D.S.); Norma.C.Garcia@ttuhsc.edu (N.G.); Edson.Ruiz@ttuhsc.edu (E.R.); 2 El Paso Children’s Hospital, El Paso, TX 79905, USA

**Keywords:** caffeine, preterm infants, discharge

## Abstract

**Background:** Apnea of prematurity (AOP) affects preterm neonates. AOP, combined with intermittent hypoxemic (IH) events frequently prolongs the length of stay. Caffeine is the preferred medication to treat AOP and may help improve IH events. There is lack of information on the safety of discharging preterm neonates home on caffeine for AOP in the literature. Our objective was to assess safety and benefits, if any, of discharging preterm infants home on caffeine. **Methods:** After IRB approval, preterm infants discharged home from the neonatal intensive care unit (NICU) on caffeine were compared with those without a discharge prescription for the period of January 2013 to December 2017. **Results:** A total of 297 infants were started on caffeine, and of those, 87 infants were discharged home on caffeine. There was no difference in length of stay between two groups. Duration of caffeine at home was 31 (28–42) days. The average cost of apnea monitor and caffeine at home per 30 days was USD 1326 and USD 50. There was no difference in number or reasons for emergency department (ED) visits or hospitalizations between two groups. **Conclusion:** AOP affects almost all preterm infants and along with intermittent hypoxemic events, and is one of the most common reasons for prolonged hospital stay. Discharging stable preterm infants home on caffeine may be safe, especially in those who are otherwise ready to be discharged and are only awaiting complete resolution of AOP/IH events.

## 1. Introduction

Apnea of prematurity (AOP) commonly occurs in preterm infants and is described as a physiological developmental disorder [1]. AOP is defined as a cessation of breathing lasting 15–20 s or longer, which may also be accompanied by bradycardia and a decline in peripheral oxygen saturation [1]. There is an inverse correlation between incidence AOP and gestational age at birth [2], occurring almost exclusively in infants born less than 37 week gestation [3,4,5]. AOP resolves by 44 weeks corrected gestation in the majority of preterm infants [2,6]. Caffeine is the preferred drug to treat AOP [7]. In addition to improved health outcomes, caffeine treatment is seen to be more cost-effective as well [8,9].

Even after the resolution of clinically apparent apneic events, intermittent hypoxemic (IH) with bradycardia and desaturations events continue to occur for a prolonged period of time [10]. Time to complete resolution of AOP/IH varies widely among preterm infants. As a result, management surrounding when to discontinue caffeine and to discharge from hospital also varies widely. No trials have been conducted to address the question of duration of treatment and when to discontinue caffeine. As a consequence, a large number of preterm infants who may otherwise be ready to be discharged remain hospitalized in need of complete resolution of AOP and or IH, thus resulting in an increased length of hospital stay, and possibly increased healthcare cost without any benefits [1,8,11]. Although some experts suggest an earlier discharge for these infants on caffeine and with or without cardiorespiratory monitoring [11,12,13], the majority of neonatal intensive care units (NICU) nationwide keep preterm infants hospitalized until 5–7 days of being apnea free. There is paucity of the literature on the safety and feasibility of discharging preterm infants home on caffeine. At our neonatal intensive care unit, we send preterm infants home on caffeine with close follow up if they are otherwise ready to be discharged. The objective of this study was to determine the safety, efficacy and economic impact of discharging stable preterm infants home with caffeine.

## 2. Materials and Methods

### 2.1. Study Population Selection

This study was reviewed by Institutional review board for the protection of human subjects at Texas Tech University Health Sciences Center at El Paso, Texas. USA and approved with a waiver of consent. IRB# E18139All infants admitted to the neonatal intensive care unit (NICU) at El Paso Children’s Hospital (EPCH) between January 2013 and December 2017, who were started on caffeine for AOP were included in the study.

Exclusion criteria included infants with severe congenital malformation, those that died before hospital discharge and those who never received caffeine or had incomplete documentation.

These infants were then sub classified into two groups, and a group of patients that were discharged home on caffeine were compared with a group of patients in whom caffeine was discontinued prior to discharge.

All preterm infants born less than 30 weeks are started on caffeine within 12 h of admission in our NICU. Preterm infants are first given a one-time caffeine bolus of 20 mg/kg and started on maintenance caffeine dose, twenty-four hours later at 6 mg/kg/day, this dose can be increased to maximum of 10 mg/kg/day, depending on clinical response. We do not check serum caffeine levels routinely. Apneic events are defined as cessation of breathing lasting more than 15–20 s, with or without associated desaturation or bradycardia [1]. All infants are monitored using the GE Dash 4000 monitor, and in addition, nurses document all AOP event with visual confirmation in EMR. An attempt to discontinue caffeine is made at around 34–35 weeks corrected gestation if the infant is without significant apneic events for 5 days consecutively and off positive pressure ventilation [7]. All preterm infants are monitored for 7 days after the discontinuation of caffeine for re-occurrence AOP [14]. If an infant continues to show signs of AOP/IH, but is otherwise stable and fulfills other milestones for safe discharge home (oral feeds, thermoregulation, assured follow up, parents must be agreeable, educated on medication administration and roomed in NICU, with or without apnea monitor), then an attending neonatologist can discharge the infant home on caffeine, with or without apnea monitor. Infants are discharged home on caffeine after observation of 5–7 days in the NICU and a dose of ten milligrams per kg per day. No dose adjustments are made post discharge. A neonatal high risk nurse conducts weekly phone call checkups on all infants discharged home on medications and or equipment. Apnea monitor recordings are reviewed by neonatologists in a follow up clinic once every two weeks, and prior to each appointment.

Infants discharged home on caffeine are seen every two weeks in high risk follow up clinic. At each follow up visit, the need for continuation of caffeine is assessed based on the caregiver’s report and a weekly review of apnea monitor recordings by neonatologists. When no events are observed or documented on apnea monitor for a minimum of 7 days, caffeine is discontinued. The apnea monitor is discontinued if, for one week following the discontinuation of caffeine, there are no significant events on the apnea monitor recording and there are no concerns by caregiver at home.

Data was collected from electronic medical records (EMR) from the time of admission through the 1st year of life. This included data from neonatal intensive care unit, outpatient neonatal high risk developmental follow up clinic and emergency department during the 1st year of life. Health insurance payer data, to assess cost, was collected for use of the apnea monitor and caffeine medication at home. 

Primary outcomes of interest were length of stay, duration of caffeine usage at home, cost of caffeine and apnea monitor use at home. Secondary outcomes included emergency department (ED) visits for morbidities, including upper respiratory infections, brief resolved unexplained events (BRUE) [15], other reasons and number of deaths during the first year of life. This data was retrieved on neonatal high risk follow up clinic notes and ED visit notes.

### 2.2. Statistical Analysis

Descriptive statistics (median, 25th and 75th percentiles for continuous variables; frequencies and percentages for categorical variables) were calculated separately by groups (Preterm infants home on caffeine vs. Preterm infants not home on caffeine). The chi-square test or Fisher’s exact test, as deemed appropriate, for categorical variables and the Mann–Whitney test (two-group comparisons) were used to assess statistical significance. A result was considered statistically significant at the *p* < 0.05. All analyses were performed using SAS version 9.4 (SAS Institute Inc., Cary, NC, USA).

## 3. Results

**Figure 1:**
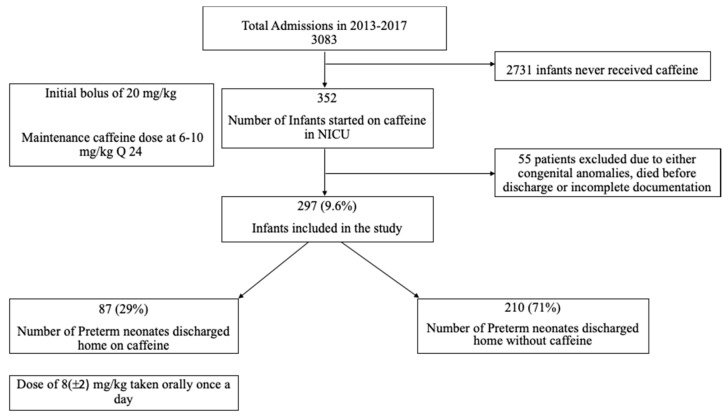
Distribution of patients.

During the study period between 2013 and 2017 a total of 3083 newborns were admitted to neonatal intensive care unit (NICU). Out of those 352 preterm newborns were started on caffeine. Two hundred and ninety-seven infants (9.6%) were included in the final analysis, and of those, 87 (29%) infants were discharged home on caffeine and 210 (71%) were discharged home without caffeine (Figure 1).

Infants who were sent home on caffeine were of lower gestation at birth (28(±3) weeks vs. 29(±2) weeks, *p* value *=* <0.05) weeks and had lower birth weight (1236(±420) grams vs. 1349(±416) grams *p* value *=* <0.03), compared to infants who were not discharged home on caffeine. The rest of the demographics were not different between the two groups (Table 1).

Infants discharged home on caffeine compared to infants not discharged on caffeine were more likely to have been diagnosed with bronchopulmonary dysplasia (BPD) [33% vs. 22%, *p* value *=* <0.05] as defined per National Institute of Child Health and Human Development (NICHD) consensus statement [16,17], and require oxygen therapy at discharge [24% vs. 13%, *p* value *=* <0.05]. There was no difference in other major morbidities or length of stay (LOS) in NICU between the both groups (Table 2).

In both groups, the first attempt to stop caffeine was made at an average gestation of 35(±2) vs. 35(±2) weeks, and both groups were discharged at similar average corrected gestation of 38(±2) vs. 38(±3) weeks. Eighty-four percent of patients sent home on caffeine, had at least one attempt to stop caffeine therapy prior to discharge.

The majority of the preterm infants discharged home on caffeine were also given an apnea event monitor recorder (91%). Preterm infants discharged home on caffeine needed a greater number of adjustments to caffeine dose during hospitalization (7(±4) vs. 5(±3), *p*-value < 0.05). Persistent apneic events were the reason for restarting caffeine in two infants. A combination of persistent apneic/bradycardia/desaturations or only bradycardia/desaturations events were reasons for restarting caffeine in 35 and 50 infants, respectively. Preterm infants discharged home on caffeine were discharged home after observation after a median of 7 (5–13) days of restarting caffeine. The duration of caffeine therapy in preterm infants discharged home on caffeine was a median of 31 (28–42) days. Caffeine was successfully discontinued at a mean corrected gestation 43(±4) weeks. The majority of the patients were on Medicaid, and the average cost of an apnea monitor and caffeine at home was USD 1326 ± 970 and USD 50 ± 34 per 30 days, based on Medicaid reimbursements of Apnea monitor use per 30 days and 10 bottles of generic caffeine citrate (60 mg/3 mL oral solution) for a 30 day supply. A greater number of infants discharged home on caffeine followed regularly in high risk clinic during first year of life (83 (95%) vs. 173 (82%), *p*-value 0.02). There was no difference in discharge weight, at 6 months nor at 1-year clinic visit between the two groups. There was no difference in the composite scores in all five domains of the ages and stages questionnaire 3rd edition performed at 6 months and at the 1-year clinic visit between the two groups. (Table 2 and Table 3).

There was no difference in number or reasons for emergency department (ED) visits or hospitalization between two groups during the first year of life. There was a total of six ED/Hospitalization visits for BRUE in both groups. Three of these infants were on caffeine at home at the time of hospitalization. There was no difference in number of deaths between the two groups as well during first year of life (Table 4).

## 4. Discussion

To the best of our knowledge, this is the largest retrospective study analyzing premature infants who were discharged home on caffeine. This retrospective study shows that discharging stable preterm infants home on caffeine may be feasible and safe when the only reason for continued NICU stay is apnea of prematurity. As was also shown in the Chimes study [6], this study confirms the fact that complete resolution of AOP/IH in more premature infants is variable and takes a long time, as shown by the fact that infants discharged home on caffeine needed caffeine until an average corrected gestation age of 43 weeks, compared to 35 weeks in the infants in whom it was stopped during hospital stay.

There are 4 million births every year in the United States [18]. Two percent of all live births are born at less than 32 weeks of gestation [3]. It is the biggest group of preterm infants that require intervention for apnea of prematurity. An important conclusion of this study is that it shows discharging stable preterm infants on caffeine therapy may be safe, without excess ED utilization or excess mortality. This study shows discharging preterm infant might also be cost effective. We show that average cost of use of apnea monitor was USD 1326 and caffeine medication at home was USD 50 per 30 days use for each individual patient. These infants were on caffeine after discharge for a median of 31 days. If they were to stay in NICU until complete resolution of AOP, that may have resulted in an increasing total cost of hospitalization and, based on previous studies [19,20,21], this likely resulted in average estimated savings of USD 77,500 per patient, basing on a conservative estimate of USD 2500 per day NICU charges.

There are several limitations to this study. First, this is a retrospective study, and several unknown factors may influence the outcomes. Besides AOP, studies conducted by Dobson [10] and Rhein et al. [22] show that some of the preterm infants continue to have intermittent hypoxemic events, even when clinically apparent apnea of prematurity have resolved. Reflux, immature suckling and pulmonary insufficiency of prematurity can all lead to some of these events. Caffeine does help increase diaphragmatic activity, stabilize the respiratory pattern, increase minute ventilation and reduce hypoxic respiratory depression, as mentioned in Dobson and Hunt 2018 [23]. The effects of these IH events earlier in life of preterm infants are well documented [24,25]. However, the long-term consequences of these IH events are unknown at this point, nevertheless, the management of these IH events remains a troublesome challenge for neonatologists. Prolonging caffeine therapy in those infants reduces the number of these hypoxemic events. As shown in the results, it was a combination of apneic/bradycardia/desaturations events that led to restarting of caffeine in preterm infants discharged home on caffeine.

Infants in caffeine group were of smaller gestation and birth weight, which makes them more likely to have AOP/IH events for longer duration, this study shows discharging these preterm infants home early on caffeine may be as safe as more mature preterm infants. Even though preterm infants in caffeine group were of lower birth weight, there was no difference in weight between the two groups at discharge, at 6 months or at 1-year clinic visit. Ages and stages questionnaire (ASQ) is a validated tool to diagnose developmental delay, and has been found to have good negative predictive value [26] and, based on the results of ASQ scores, our study suggests no adverse effects of prolonging caffeine therapy as outpatient.

This study also does not account for other common reasons for delayed discharge, including time to achieve the ability to take feeds orally, thermoregulation etc. In 16% of patients sent home on caffeine, there was never any attempt to stop it prior to discharge. The reasons for this were unclear upon reviewing in patient notes, other than neonatologist’s preference. Ninety five percent of the infants sent home on caffeine and 82% of patient discharged home without caffeine did follow up regularly in a high risk neonatal clinic, and at every clinic visit, all interim healthcare interactions are documented. Even then, some of the morbidity data, related to healthcare utilization during first year of life, may not be complete as our hospital is in the city of El Paso, Texas, which is along an international border between United States and Mexico. As a consequence, some patients may have received care on the other side of the border during the first year of life.

We acknowledge that there is lack of correlation between electronic monitor readings and nursing documentation for the timely and accurate assessment of AOP, as shown in previous studies. The majority of NICUs nationwide continue to use a combination of electronic monitoring with nursing documentation of events to diagnose and treat AOP. Hence, the results of our study may be generalizable. A greater number of infants in the caffeine group were discharged on home oxygen. Infants discharged home on oxygen and caffeine were monitored via pulse oximetry, in addition to an apnea monitor. These patients were followed in a high risk follow clinic once in two weeks and were managed by neonatologists, unless they were tracheostomy dependent, which were excluded from this analysis. Discharging infants on home oxygen could potentially add to outpatient care cost. We did not follow number of days an infant was on oxygen at home; however, Medicaid reimbursement for home oxygen and pulse oximetry in our area are on an average USD 350 per month use, so the effect on overall cost are likely negligible.

The majority of the preterm infants in the caffeine group were discharged home on apnea monitor, and there are currently no data to support the routine use of home cardiorespiratory monitoring. As mentioned in the AAP clinical report [27] and by other authors [28], we do not suggest the routine use of home CR monitoring, with only few exceptions with adequate parental counselling and training.

One aspect that is often overlooked is that the parents of preterm infants often experience loss of their parental role and prolonged hospitalization disrupts parent-infant bonding [29]. To be able to discharge infants home sooner may benefit the social and emotional well-being of caregivers.

Larger prospective randomized trials are needed to confirm these results. A prospective randomized trial comparing moderately late preterm infants randomly assigned to receive caffeine at home vs. not, is underway (MoCHA trial), which will help shed more light on this issue.

## 5. Conclusions

Apnea of prematurity affects almost all preterm infants universally. Apnea of prematurity along with intermittent hypoxemic events is one of the most common reasons for prolonged hospital stay. Discharging stable preterm infants home on caffeine may be safe, especially in those who are awaiting complete resolution of AOP/IH events and are otherwise ready to go home. This has the potential to shorten the length of stay and decrease overall healthcare cost.

## Figures and Tables

**Table 1:** Maternal and neonatal characteristics.

Maternal & Newborn Characteristics	Preterm Infants Home on Caffeine (87)	Preterm Infants Home without Caffeine (210)	*p* Value
Gestational age, weeks ^a^	28 ± 3	29 ± 2	<0.05 ^Ω^
Birth weight, grams ^a^	1236 ± 420	1349 ± 416	0.03 ^Ω^
Hispanic, n (%)	64(74)	143(68)	NS
Vaginal, n (%)	32(37)	71(34)	NS
Male, n (%)	51(58)	98(47)	NS
Apgar score, n ^b^, min			
1	6(3–7)	6(4–7)	NS
5	7(6–8)	7(6–8)	NS
Apgar ≤ 5 at 5 min, n (%)	16(18)	31(15)	NS
Maternal Prenatal care n (%)	84(96)	196(93)	NS
SGA n (%)	12(14)	27(13)	NS
Antenatal Steroids, n (%)	66(76)	164(79)	NS

^a^—mean ± SD; ^b^—median (interquartile range); ^Ω^–Student unpaired *t*-test; rest by Fisher exact test. NS–Not significant. SGA–Small for Gestational age.

**Table 2:** Neonatal outcome in hospital.

In Hospital Morbidity	Preterm Infants Home on Caffeine (87)	Preterm Infants Home without Caffeine (210)	*p* Value
Respiratory distress syndrome, n (%) Given surfactant	64(74)	135(64)	NS
No. of days of mechanical Ventilation ^a^	19 ± 27	19 ± 32	NS ^Ω^
BPD, n (%)	29(33)	46(22)	0.05
Oxygen at discharge *, n (%)	21(24)	27(13)	0.02
Intraventricular hemorrhage (IVH)			
Any IVH, n (%)	57(72)	152(66)	NS
Severe IVH (grade III or IV), n (%)	8(9)	8(4)	NS
Retinopathy of prematurity (ROP)requiring anti vascular endothelial growth factor (VEGF) treatment, n (%)	4(4.5)	10(4.7)	NS
Necrotizing enterocolitis, any stage, n (%)	1(1)	14(6)	NS
Discharge weight (grams) ^a^	2654 ± 493	2625 ± 632	NS ^Ω^
Discharge head circumference ^a^	33 ± 1.7	33 ± 1.5	NS ^Ω^
Length of Stay, days ^a^	68 ± 30	62 ± 36	NS ^Ω^

^a^–mean ± SD; ^Ω^—Student unpaired *t*-test; rest by Fisher exact test; * infants discharged home on low flow nasal cannula oxygen; bronchopulmonary dysplasia (BPD): defined per NICHD consensus statement as need of oxygen support at 36 weeks of postmenstrual age (PMA).

**Table 3:** Outcome after discharge.

Parameters at Discharge and During First Year of Life	Preterm Infants Home on Caffeine (87)	Preterm Infants Home without Caffeine (210)	*p* Value
Apnea Monitor at discharge, n (%)	80(91)	1(0.04)	-
Number of days on Caffeine till first attempt to stop ^a^	47 ± 28	39 ± 28	0.02 ^Ω^
Ever an attempt to stop caffeine, n (%)	73(84)	210(100)	<0.05
Corrected gestation at first attempt to stop in nicu ^a^	35 ± 3	35 ± 3	NS
Gestation at discharge ^a^	38 ± 2	38 ± 4	NS
No. of adjustments to dose made during hospitalization ^a^	7 ± 4	5 ± 3	<0.05 ^Ω^
Median days to restarting caffeine after initial attempt	5(3–9)	-	-
Reasons for Restarting Caffeine		-	
No. of infants with persistent Apneic events	2		
No. of infants with combination of persistent Apneic, Bradycardia and Desaturations events	35		
No. of infants with combination of persistent bradycardia and events	54		
No. of days from Caffeine Stop to discharge ^b^	-	16(10–25)	-
Dose (mg/kg/day)of Caffeine at discharge ^a^	8 ± 2	-	-
No. of days monitored in NICU from caffeine restart to discharge	7(5–13)	-	-
No. of days on caffeine post discharge ^b^	31(28–42)	-	-
Cost of Apnea monitor use post discharge in dollars USD 750 per 30 days (Medicaid reimbursement) ^a^	USD 1326 ± 970	-	-
Cost of Caffeine at home in dollars ^a^	USD 50 ± 34	-	-
Corrected gestational age caffeine stopped after discharge ^a^	43 ± 4	-	
No. of days on apnea monitor after caffeine stop at home ^b^	31(11–36)	-	-
No. Infants with follow up in Neonatal follow up clinic, n (%)	83(95)	173(82)	0.02 ^Ω^
Weight (kg) at 6 months clinic visit ^a^	6.5 ± 1.2	6.4 ± 1	NS
Weight (kg) at 1 year clinic visit ^a^	8 ± 1	8.3 ± 1	NS
Scores in 5 domains of Ages and Stages Questionnaire			
At 6 months clinic visit ^a^			
Communication	53 ± 9	55 ± 6	NS ^Ω^
Gross motor	50 ± 11	48 ± 13	NS ^Ω^
Fine motor	50 ± 12	51 ± 11	NS ^Ω^
Problem solving	53 ± 11	53 ± 10	NS ^Ω^
Personal social	51 ± 10	49 ± 12	NS ^Ω^
At 1-year clinic visit ^a^			
Communication	42 ± 16	47 ± 13	NS ^Ω^
Gross motor	47 ± 20	48 ± 16	NS ^Ω^
Fine motor	47 ± 16	51 ± 12	NS ^Ω^
Problem solving	46 ± 16	50 ± 19	NS ^Ω^
Personal social	41 ± 16	47 ± 19	NS ^Ω^

^a^—mean ± SD; ^b^—median (interquartile range); ^Ω^—Student unpaired *t*-test; Fisher exact test used for rest of the parameters.

**Table 4:** Emergency department (ED) visits and hospitalization data.

Parameters during First Year of Life Post NICU Discharge	Preterm Infants Home on Caffeine (87)	Preterm Infants Home without Caffeine (210)	*p* Value *
**Infant with ED Visits Post Discharge**			
No. of infants with ED visit after discharge in first year of life, n (%)	26(30)	67(32)	NS
Reasons and number for ED visits within first year of life after discharging home			
BRUE, n (% of total no. of infant with ED visit)	6(23)	6(9)	NS
Respiratory disease, n (%of total no. of infant with ED visit)	15(57)	44(65)	NS
Fever, n(%of total no. of infant with ED visit)	6(2)	12(2)	NS
AGE, n(% of total no. of infant with ED visit)	4(2)	20(30)	NS
Rash, n (% of total no. of infant with ED visit)	0(0)	4(1)	NS
Other, n (% of total no. of infant with ED visit)	25(96)	50(75)	NS
Total number of ED visits, n	56	136	NS
Ratio of Total number of ED visit to total infants visiting ED	2.1	2	
**Infants Hospitalized Post Discharge**			
Total number of infants hospitalized after discharge in first year of life, n (%)	10(11)	20(10)	NS
Respiratory disease, n (% of total no. of infant Hospitalized)	2(20)	6(30)	NS
Fever, n (% of total no. of infant Hospitalized)	1(10)	1(5)	NS
AGE, n (% of total no. of infant Hospitalized)	0	3(15)	NS
Rash, n (% of total no. of infant Hospitalized)	0	0	NS
Other, n (%of total no. of infant Hospitalized)	1(10)	6(30)	NS
**Details of Brief Resolved Unexplained Events (BRUE/ALTE)**			
	**Preterm infants home on caffeine**	**Preterm infants home without caffeine**	
Total number of infants	6	6	
ED visits only	0	4	
On caffeine at home when ED/Hospitalized	3		
Deaths in First year of life, n (% of all infants)	0	3(1.4)	NS
Cause of death		SIDS in all three	

BRUE: brief resolved unexplained event (previously known as Apparent life threatening event (ALTE); AGE: acute gastroenteritis; * Fisher exact test used for statistics.

## Abbreviations

AOP	Apnea of prematurity
LOS	Length of stay
NICU	Neonatal intensive care unit
CR	Cardiorespiratory monitoring
EPCH	El Paso Children’s Hospital
BRUE	Brief resolved unexplained event
ED	Emergency department
BPD	Bronchopulmonary Dysplasia
AGE	Acute Gastroenteritis
IH	Intermittent Hypoxemic Events
ASQ	Ages and Stages Questionnaire 3rd edition
